# Negative Appendectomy Rates and Their Correlation With the Use of Histopathology: A Clinical Audit

**DOI:** 10.7759/cureus.101587

**Published:** 2026-01-15

**Authors:** Jamshid Khan, Yasir Hakim, Aalia Amjad, Asad Munir, Syed M Najeeb, Hamza Khan

**Affiliations:** 1 General Surgery, Khyber Medical College, Peshawar, PAK; 2 General Surgery, Khyber Medical University, Peshawar, PAK

**Keywords:** appendicitis, clinical audit, diagnostic error, histopathology, negative appendectomy

## Abstract

Objectives

To analyze the rate of negative appendectomies in a tertiary care hospital through assessment of the clinical diagnostic skills of surgical residents while keeping the histopathology report as the gold standard.

Methodology

This clinical audit was conducted at the Surgical ‘C’ Unit of Khyber Teaching Hospital in Peshawar. The study spanned a six-month period (1st of July 2024 to 31st of December 2024). A sample size of 100 patients (both genders) was selected through a non-probability consecutive sampling technique. All patients with a histopathological report of a normal appendix (peri-appendicitis) and appendicitis were identified and included. All the data collected through the fulfillment of proformas were added to a Microsoft Excel^®^ (Microsoft, Redmond, WA, USA) sheet and transferred to SPSS version 23.0 (IBM Corp., Armonk, NY, USA) for data analysis and verification. The results are illustrated in the form of description tables, charts, and figures.

Results

Out of 100 patients who were included in the audit cycle, during the six-month period, 37 (37%) were female patients and 63 (63%) were male patients. Most patients were 20-30 years of age with a mean age of 22.4 ± 0.871. According to the histopathology reports obtained, 89 (89%) patients had acute appendicitis, 6 (6%) patients had gangrenous appendicitis, and 5 (5%) patients had periappendicitis (normal appendix).

Conclusion

The negative appendectomy rate was less than 5% in this study. Female and younger patients (age ≤ 35 years) should be of great concern because they may increase the negative appendectomy rate.

## Introduction

Open appendectomy is the first surgical procedure a postgraduate resident is expected to learn and accomplish during surgical residency, making it the most common emergency surgical procedure performed worldwide. Acute appendicitis is considered as one of the most common causes for pain in the lower right quadrant of the abdomen [1,2]. Some studies have suggested an overall lifetime risk of 7 to 8%, with 8.6% in men and 6.7% in women [3]. Due to complications related to acute appendicitis, uncomplicated cases of acute appendicitis have been treated with a conservative approach through application of antibiotics, pain killers, and intravenous fluids [4]. However, the gold standard for acute appendicitis remains appendectomy in the form of an open approach or a laparoscopic approach because randomized trials have illustrated incomplete response with conservative management for the majority of cases [5].

Just like any other surgical procedure, appendectomy is also associated with adverse events, and one of the common complications is a negative appendectomy (NA). The rates of negative appendectomy (NARs) worldwide have varied, as shown by multiple trials with their respective percentages: 4% [6], 10% [7], and 45% [8]. An NAR is usually defined as no macroscopic or histological evidence of inflammation where there is no infiltration of the mucosal wall by inflammatory cells, particularly polymorphonuclear leucocytes, lymphocytes, or plasma cells. NA has an impact on the financial burden of hospital resources, increases the morbidity of patients, and prolongs the hospital stay as well as exposure to anesthesia complications [9,10].

Historically, the diagnosis of acute appendicitis has been achieved through clinical examination. However, the advent of radiological imaging techniques such as ultrasound [11] and CT scan [12] has reduced the false-negative rates from clinical examination. Computed tomography of the abdomen and pelvis has reduced the NAR to less than 10% [13], which is regarded as an acceptable percentage worldwide. The fact that this modality is now readily available, with a relatively lower risk of radiation-induced injury, has further advocated the reliability of this investigation. However, there is debate over the ideal imaging protocol for lowering the NAR without increasing the rate of missed appendicitis.

The World Society of Emergency Surgery (WSES) has published guidelines on various topics related to emergency surgery, including acute appendicitis. Initially introduced in 2015 and recently upgraded in 2020, some aspects related to acute appendicitis that have been covered in these guidelines include diagnostic accuracy, non-operative management, intraoperative grading, and proper selection of surgical candidates. The aim of this audit is to evaluate the NAR in our surgical unit as well as educate the postgraduate surgical residents about the WSES guidelines and how these guidelines can be applied in clinical practice to further reduce the NAR. 

Objectives

1. Primarily to evaluate the NAR in our surgical unit. 

2. Educate the postgraduate surgical residents about the WSES guidelines. 

## Materials and methods

After attaining approval from the Hospital Ethical and Audit Review Committee, the clinical audit began with a sample size of 100 patients (both genders) who were selected through a non-probability consecutive sampling technique at the Surgical ''C'' Unit, Khyber Teaching Hospital, Peshawar. The study duration was six months (from 1st July 2024 to 31st December 2024). All patients, irrespective of age and belonging to both genders, with a histopathological report of a normal appendix and appendicitis were identified and included, while elective appendectomies in the form of interval or accidental appendectomies, patients who underwent appendectomy for other reasons without preoperative diagnosis of acute appendicitis, and patients who had no pathological reports were excluded from the study. Medical files of all the patients were obtained from the record room for all the needed information. The following demographic parameters were recorded: name, age, gender, address, associated symptoms, and laboratory findings to calculate the appendicitis inflammatory response (AIR) score, education, date of admission, date of discharge, medical record number, contact number, hemodynamic indicators, and histopathology reports.

A total of 100 patients were selected through a non-probability consecutive sampling technique as per our inclusion criteria. As soon as we reached our target number of appendectomies, we did not include any more cases and proceeded with data entry and analysis, which utilized most of our study duration. An NA means surgery was done to remove the appendix because appendicitis was suspected, but the appendix was actually healthy or showed no signs of inflammation when examined during surgery, and especially after histopathology analysis. All cases were performed through an open technique by the senior-most resident of our unit. The rationale of intraoperative decision-making criteria was based on signs of inflammation, such as the presence of peritoneal fluid in the peri-appendicular space, elongation of the appendix, as well as congestion and edema. Both ultrasound and CT scan were employed as imaging modalities in subclinical conditions, and CT was done on the recommendations of the consultant during morning rounds. 

**Figure 1:**
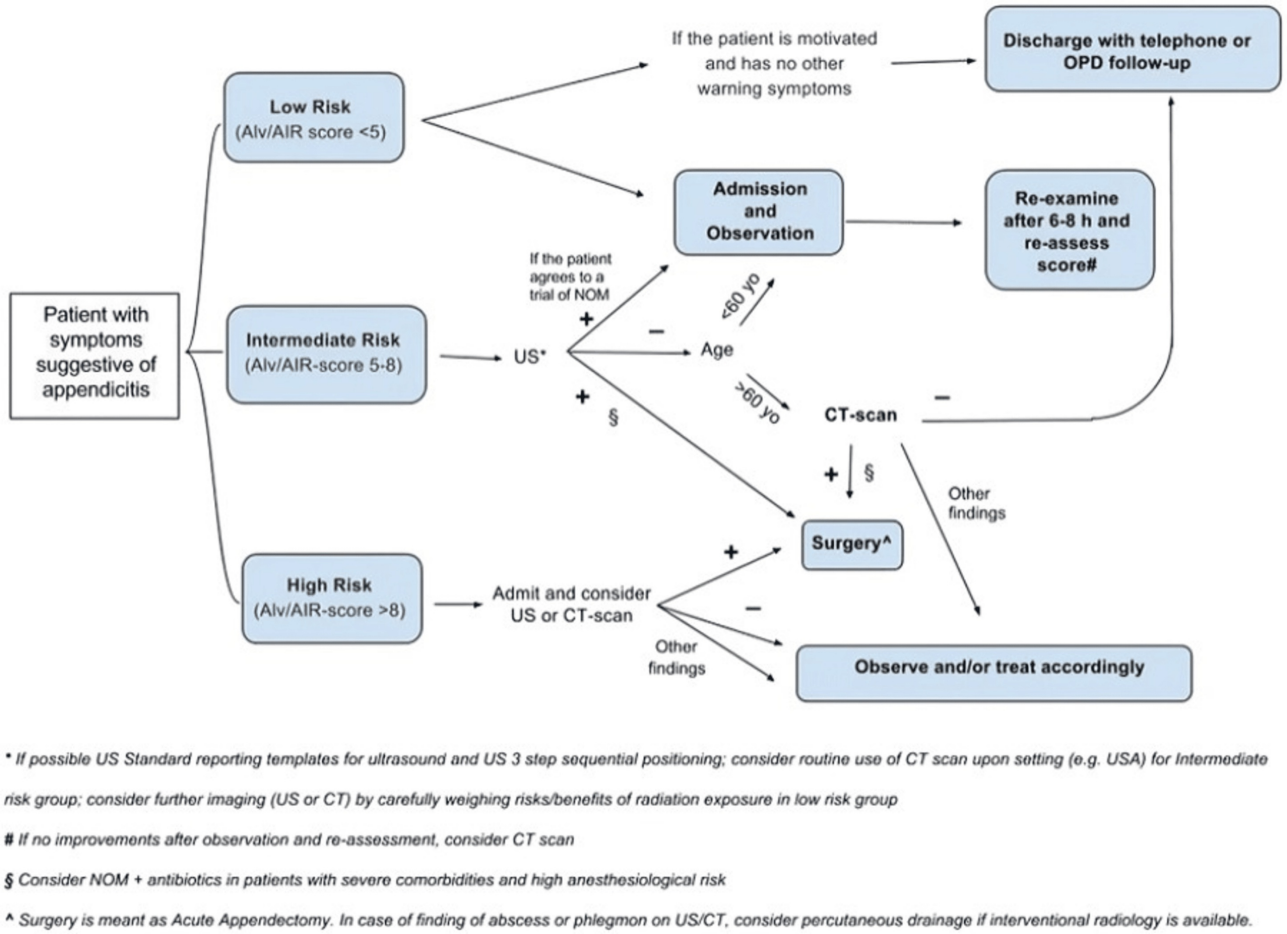
Practical WSES algorithm for diagnosis and treatment of patients with suspected acute appendicitis. WSES, World Society of Emergency Surgery; NOM, non operative management; Alv, Alvarado score; AIR, appendicitis inflammatory response; US, ultrasound; OPD, outpatient department; AAS, adult appendicitis score. The content/image is published under a Creative Commons License. Source: Di Saverio et al., 2020 [16].

The Alvarado score was calculated using the following parameters with scores given in brackets: migratory pain (1), tenderness in the right iliac fossa (2), rebound tenderness (1), nausea (1), anorexia (1), raised temperature (1), leukocytosis (2) (white blood cell ≥10,000), and shift of white blood cell count to the left (neutrophil ≥75%) (1) [14]. Appendicitis inflammatory response score (AIR) was calculated by using the following parameters with their respective scores: vomiting (1), right iliac fossa pain (1), rebound tenderness (2), raised temperature (1), polymorphonuclear leukocytes >85% (2), WBC count >×10⁹/L (1), and CRP level (mg/L) >10-49 (1) [15]. The operative findings and outcomes following surgery were also recorded. All the data were collected through the fulfillment of proformas, transferred to a Microsoft Excel^®^ (Microsoft, Redmond, WA, USA) sheet, and data analysis was done using SPSS version 23.0 (IBM Corp., Armonk, NY, USA). Quantitative variables were presented in the form of frequencies and percentages, while qualitative variables were illustrated in the form of means and standard deviation. The results were compared with similar trials related to the subject and are illustrated in the form of discussion, tables, and figures. Also, the purpose of this study is to introduce the surgical residents to WSES guidelines, as shown in Figure 1 [16], on the accurate diagnosis of acute appendicitis in order to further control the rate of negative appendectomies.

## Results

Out of 100 patients, during the six-month period, most patients were in the range of 21-30 years of age (n = 45, 45%) with a mean age of 22.4 ± 0.87 years. The average length of hospital stay was 1.21 ± 0.05 days, ranging from a minimum of one day to a maximum of three days, depending upon the case and the clinical condition of the patient. The mean Alvarado score was 6.6 ± 0.77, ranging from a maximum value of 8 to a minimum value of 5 (Tables 1, 2). In the audit cycle, 37 (37%) were female patients and 63 (63%) were male patients. According to the histopathology reports obtained, 89 (89%) patients had acute appendicitis, 6 (6%) patients had gangrenous appendicitis, and 5 (5%) patients had peri-appendicitis (normal appendix). The AIR score was utilized: 89 (89%) patients were labeled as intermediate risk, 6 (6%) patients as high risk, and 5 (5%) patients as low risk. Details are shared in Table 3. 

**Table 1:** Mean and standard deviation of quantitative variables.

Variable	Mean	Standard deviation
Age (years)	22.4	0.87
Average length of hospital stay (days)	1.21	0.50
Alvarado score	6.6	0.77

**Table 2:** Frequency and percentage of age groups, Alvarado score, and length of hospital stay in days.

Age (years)	Frequency	Percentage
1-20	15	15
21-30	45	45
31-40	20	20
41-50	20	20
Alvarado score		
5	10	10
6	29	29
7	54	54
8	7	7
Length of hospital stay (days)		
1	74	74
2	12	12
3	14	14

**Table 3:** Frequency and percentage of gender, histopathology report, and AIR/AAS scores. AIR, appendicitis inflammatory response.

Gender	Frequency	Percentage
Male	37	37
Female	63	63
Histopathology report		
Acute appendicitis	89	89
Peri-appendicitis	5	5
Gangrenous appendicitis	6	6
AIR score		
Intermediate	89	89
Low	5	5
High	6	6

## Discussion

Patients who undergo an NA are exposed to all the surgical complications related to any procedure that involves intervention, which include wound infections, hematoma formation, seroma formation, iatrogenic injuries, exposure to anesthesia, as well as putting a burden on hospital resources. Therefore, the problem of NA is not uncommon. The reason behind this surgical dilemma is that symptoms related to many conditions resemble those of acute appendicitis, which include Crohn's disease, terminal ileitis, typhoid, and gynecological diseases [17]. Concerns have been raised about decreasing the NAR in order to overcome these problems. Therefore, it is of paramount importance to consider that every possible effort should be made to achieve a correct diagnosis of acute appendicitis. 

According to the histopathology reports obtained in this study, only 5% of the patients had a normal appendix. This proportion is globally acceptable and comparable to multicentric studies with the purpose of determining an NAR [18]. The NAR is heavily dependent upon the medical facilities provided at institutions as well as the clinical experience of surgical residents. Postgraduate surgical residents have often been regarded as safe and effective in the diagnosis and management of acute appendicitis [19]. All six patients who had an NA were female patients in this study. This finding is supported by studies [20,21] done on the topic, citing the reason that some gynecological diseases mimic acute appendicitis, hence leading to a higher NAR in female patients. On the contrary, Noureldin et al. [22] depicted that both male and female patients were equally affected in their paper. This equal distribution has eliminated the potential gender bias in some trials. 

As far as the factor of age is concerned, five out of six patients with NA in our study were between the ages of 25 and 35. Hence, younger patients were more prone to undergo an NA as compared to elderly patients. According to WSES guidelines, patients with suspected acute appendicitis on ultrasound but who are above the age of 40 should undergo computed tomography (CT) of the abdomen and pelvis to have a more definitive diagnosis. A landmark study published by Rao et al. presented probably the earliest evidence that CT scan was effective in decreasing the NAR from 20% to 7% [23]. A history of loose motions and abdominal colic may mislead the surgeon toward a pelvic appendicitis, hence leading to an NA. These symptoms may be similar to enterocolitis or irritable bowel syndrome (IBS). When diagnosing acute appendicitis, clinicians should be cautious if patients experience abdominal discomfort and diarrhea [24,25].

However, despite the recent advances in biochemical and imaging techniques, a proper history and clinical examination remain the cornerstone for the diagnosis of acute appendicitis. Laboratory investigations in the form of a total leukocytic count and a C-reactive protein are considered the most specific biochemical investigations [26,27]. Various decision tools, including clinical algorithms, checklists, and scoring systems, exist to help substantiate the diagnosis of acute appendicitis. In our study, the AIR [28] revealed that 89% of the patients fell in the intermediate-risk group, 6% in the low-risk group, and 5% in the high-risk group. According to the WSES guidelines, patients with strong signs and symptoms and high risk of appendicitis according to AIR score/Alvarado score/and younger than 40 years old may not require cross-sectional pre-operative imaging (i.e., CT scan); however, those patients with a mild to moderate clinical suspicion of acute appendicitis are advised to undergo radiological investigations in order to avoid the chance of undergoing an NA. 

Study limitations

This study has several limitations that should be considered when interpreting the findings. First, as a clinical audit, it was observational in nature and therefore unable to establish causality between diagnostic practices and NARs. The results primarily reflect local practice patterns and may not be fully generalizable to other institutions with different patient populations or diagnostic resources.

Second, the audit relied on retrospective data collection, which is subject to incomplete documentation and potential information bias. Details regarding clinical decision-making, surgeon experience, and the rationale for proceeding to surgery may not have been consistently recorded.

Third, the study did not systematically evaluate the use or accuracy of preoperative imaging or clinical scoring systems. As a result, the direct impact of these diagnostic tools on NARs could not be assessed. Similarly, patient-related factors such as symptom duration and comorbidities were not analyzed in detail.

Fourth, histopathological interpretation itself may be subject to interobserver variability, particularly in cases of early or resolving appendicitis. While histopathology is considered the gold standard, minor inflammatory changes may be interpreted differently across pathologists.

Finally, the cost-effectiveness analysis of routine histopathological examination was beyond the scope of this audit. Although diagnostic value was demonstrated, future studies should assess the economic implications of mandatory histopathology in appendectomy specimens.

## Conclusions

NA remains a key challenge in treating patients with acute right lower abdomen discomfort. This study found an NAR of less than 5%. Female and younger patients (age ≤35 years) pose a greater risk for NA. The use of CT scans in certain circumstances may further reduce NAR, while it may not be possible to totally eliminate it. A strong index of suspicion for other complicating diseases, as well as meticulous clinical skills from experienced doctors, may help to avoid an NA.
